# Supplementary material for: escheR: Unified multi-dimensional visualizations with Gestalt principles
Source: bioRxiv. 2023 Jun 8:2023.03.18.533302. Originally published 2023 Mar 23. Preprint. [Version 2] doi: 10.1101/2023.03.18.533302 (PMC10055209; doi:10.1101/2023.03.18.533302)
Supplement: Supplement 1 [file media-1.pdf]

Supplementary Materials

escheR: Uni ed multi-dimensional visualizations with Gestalt principles

Boyi Guo, Stephanie C. Hicks

Correspondence to shicks19@jhu edu

Contents

1. **Supplemental Figures S1.**

Supplemental Figures

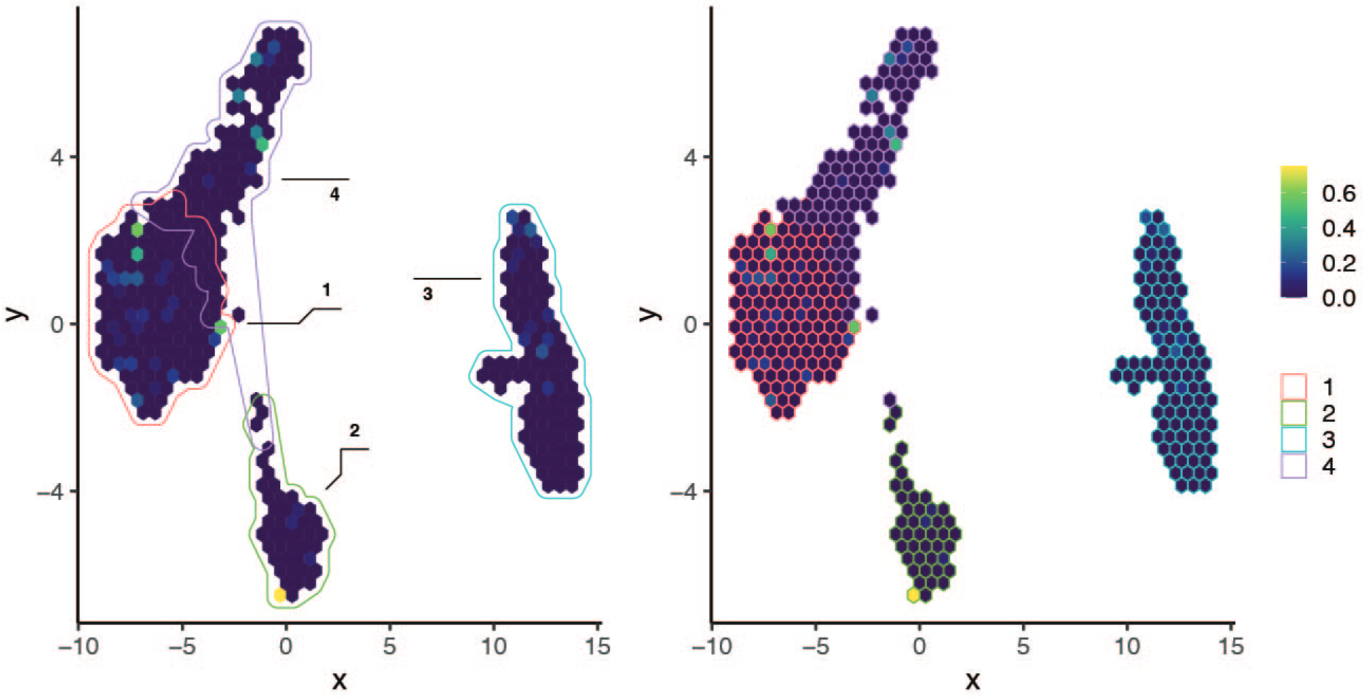

Supplementary Figure S1: **`escheR` enables multi dimensional embedding visualizations**. The gene expression of *POMGNT* among peripheral blood mononuclear cells [29] under the UMAP representation. (**A**) The `schex` R/Bioconductor package uses color-coded convex hulls to annotate data-driven cell types, creating confusion when interpreting hexagons in overlapping hulls. (**B**) `escheR` plots hexagon-specific membership to improve interpretability.
